# High-dose rapamycin exerts a temporary impact on *T. reesei* RUT-C30 through gene *trFKBP12*

**DOI:** 10.1186/s13068-021-01926-w

**Published:** 2021-03-26

**Authors:** Ai-Ping Pang, Haiyan Wang, Funing Zhang, Xin Hu, Fu-Gen Wu, Zhihua Zhou, Wei Wang, Zuhong Lu, Fengming Lin

**Affiliations:** 1 State Key Laboratory of Bioelectronics, School of Biological Science and Medical Engineering, Southeast University, Nanjing, China; 2 Key Laboratory of Synthetic Biology, Institute of Plant Physiology and Ecology, Shanghai Institutes for Biological Sciences, Chinese Academy of Sciences, Shanghai, China; 3 State Key Lab of Bioreactor Engineering, New World Institute of Biotechnology, East China University of Science and Technology, Shanghai, China

## Abstract

**Background:**

Knowledge with respect to regulatory systems for cellulase production is prerequisite for exploitation of such regulatory networks to increase cellulase production, improve fermentation efficiency and reduce the relevant production cost. The target of rapamycin (TOR) signaling pathway is considered as a central signaling hub coordinating eukaryotic cell growth and metabolism with environmental inputs. However, how and to what extent the TOR signaling pathway and rapamycin are involved in cellulase production remain elusive.

**Result:**

At the early fermentation stage, high-dose rapamycin (100 μM) caused a temporary inhibition effect on cellulase production, cell growth and sporulation of *Trichoderma reesei* RUT-C30 independently of the carbon sources, and specifically caused a tentative morphology defect in RUT-C30 grown on cellulose. On the contrary, the lipid content of *T. reesei* RUT-C30 was not affected by rapamycin. Accordingly, the transcriptional levels of genes involved in the cellulase production were downregulated notably with the addition of rapamycin. Although the mRNA levels of the putative rapamycin receptor trFKBP12 was upregulated significantly by rapamycin, gene *trTOR* (the downstream effector of the rapamycin–FKBP12 complex) and genes associated with the TOR signaling pathways were not changed markedly. With the deletion of gene *trFKBP12*, there is no impact of rapamycin on cellulase production, indicating that *trFKBP12* mediates the observed temporary inhibition effect of rapamycin.

**Conclusion:**

Our study shows for the first time that only high-concentration rapamycin induced a transient impact on *T. reesei* RUT-C30 at its early cultivation stage, demonstrating *T. reesei* RUT-C30 is highly resistant to rapamycin, probably due to that trTOR and its related signaling pathways were not that sensitive to rapamycin. This temporary influence of rapamycin was facilitated by gene *trFKBP12*. These findings add to our knowledge on the roles of rapamycin and the TOR signaling pathways play in *T. reesei*.

**Supplementary Information:**

The online version contains supplementary material available at 10.1186/s13068-021-01926-w.

## Background

Cellulolytic fungi like *Neurospora crassa* and *Trichoderma reesei* are involved in the degradation of plant biomass and play important roles in ecosystems [1, 2]. These fungi have evolved an excellent capability to secret (hemi)cellulase to convert insoluble polysaccharides into fermentable sugars for surviving under lignocellulolytic conditions. The (hemi)cellulase should be produced in a strictly controlled way. Otherwise, too much cellular resources are directed towards protein synthesis, which is detrimental for cell survival [1]. Therefore, cellulolytic fungi have developed a complex regulatory machinery to coordinate nutrients for growth and hydrolytic enzyme production. For instance, the cellulase production of *T. reesei* is regulated in response to various environmental stresses such as light [3, 4], organic solvents [5], and metal ions [6]. Several signal pathways like mitogen-activated protein kinase (MAPK) pathways [7], Ca^2+^-responsive signaling pathway [8] and light regulation pathway [3] have been reported to be involved in the regulation of cellulase production. A better understanding of regulatory systems for cellulase production is prerequisite for exploitation of such regulatory networks to increase enzyme secretion, improve fermentation efficiency and reduce the enzyme production cost.

The target of rapamycin (TOR) signaling pathway is regarded as a central signaling hub integrating cell growth and metabolism with environmental inputs, including nutrients and growth factors in eukaryotes [9]. The TOR signaling network responds to signals such as nutritional state, cellular energy state, and growth factors to orchestrate cellular growth, proliferation, and stress responses. In response to nutrients, it activates anabolic processes like protein, lipid and nucleotide synthesis, and suppresses catabolic processes such as autophagy, thus promoting cell growth. Several studies have reported that the TOR signaling pathway plays a part in regulating cellulase production. Gene *sch9* is a critical component of the TOR signaling pathway in *Saccharomyces cerevisiae*. Deletion of its homolog *stk-10* in *N. crassa* severely decreased cellulase production [10]. Similarly, loss of *schA* (the homolog of *stk-10*) in *Aspergillus nidulans* caused defects in protein production and cellulolytic enzyme activities [11]. Also, knockdown of *sch9* in *T. reesei* TU-6 slightly compromised the cellulase production [12]. Despite these preliminary studies, how and to what extent the TOR signaling pathway is involved in cellulase production remains elusive. Even worse, the relevant study of the TOR signaling pathway in filamentous fungi is lacking, though it has been extensively studied in yeast *S. cerevisiae* and *Schizosaccharomyces pombe* [13, 14], mammalian cells [15], and plant cells [16, 17].

The TOR kinases were first identified in *S. cerevisiae* as the targets of rapamycin. Rapamycin, a lipophilic macrocyclic lactone isolated from *Streptomyces hygroscopicus* in the early 1970s [18], displays antifungal, anticancer and immunosuppressive activities [9]. Rapamycin and its derivatives, which are called “rapalogues”, have been applied in clinic to inhibit tumor growth and prevent organ rejection [9]. When diffusing into the cell, rapamycin forms a complex with the peptidyl-prolyl *cis/trans* isomerase FKBP12, which subsequently binds to the TOR kinases and inhibits their functions [14, 19, 20]. Both FKBP12 and TOR kinases are conserved in eukaryotic organisms from fungi to human [21–29]. Rapamycin is an indispensable tool for studying the role of the TOR signaling pathway in organisms. However, the impact of rapamycin on *T. reesei* and cellulase production in filamentous fungi has not been reported, as far as we know.

As a first step toward understanding the role of the TOR pathway in cellulase production, the impact of rapamycin on *T. reesei* RUT-C30 grown on different carbon sources was investigated in terms of cellulase production, morphology, cell growth, sporulation and lipid content. *T. reesei* RUT-C30 was insensitive to rapamycin and only a temporary effect was observed with high concentration of rapamycin at the early cultivation stage, including decreased cellulase production, cell growth and sporulation, and altered cell morphology. The molecular mechanism behind this phenomenon was explored by comparative transcriptional profiling and gene knockout of *trFKBP12*.

## Results

### Rapamycin at high concentration temporarily inhibits cellulase production

**Fig. 1:**
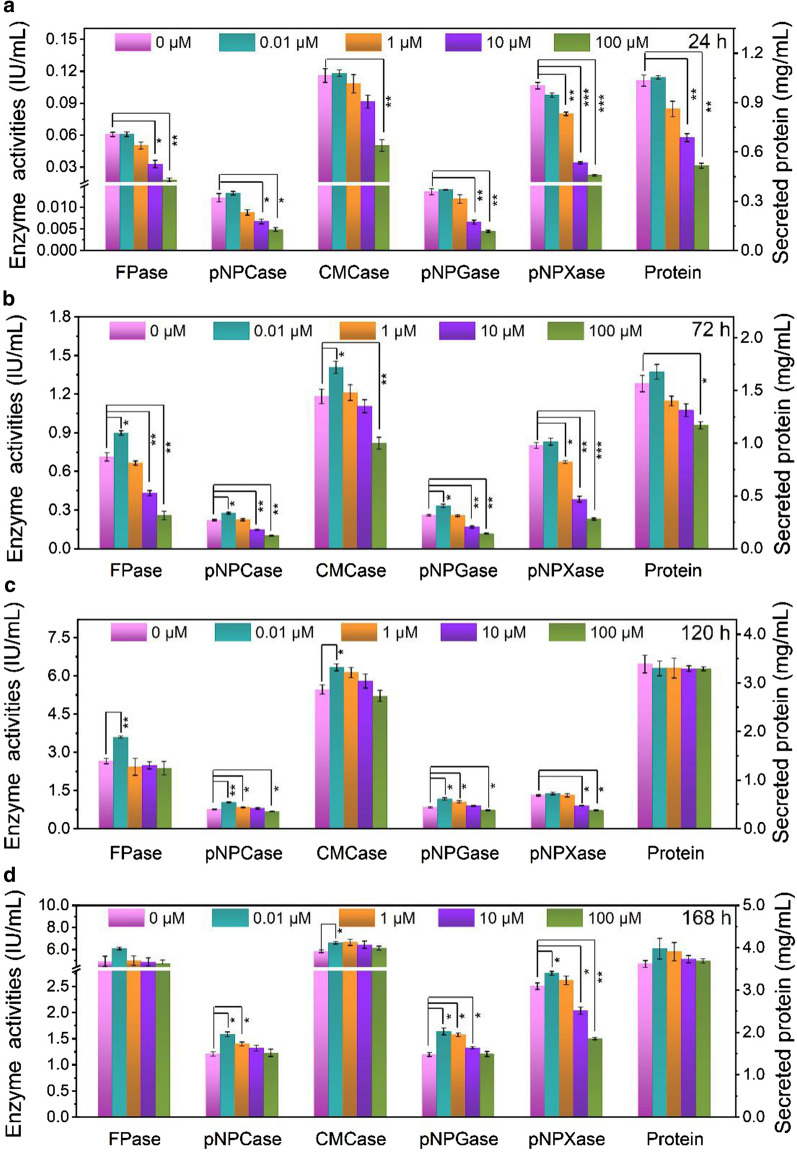
The (hemi)cellulase activities and protein secretion of *T. reesei* RUT-C30 cultured in TMM + 2% cellulose with different concentrations of rapamycin at 24 h (**a**), 72 h (**b**), 120 h (**c**), and 168 h (**d**), respectively. FPase: the filter paper activity; pNPCase: the CBH activity; CMCase: the CMC activity; pNPGase: the β-glucosidase activity; pNPXase: the β-xylosidase activity; Secreted protein: secreted protein concentration. Data are represented as the mean of three independent experiments and error bars express the standard. Asterisks indicate significant differences (*p < 0.05, **p < 0.01, ***p < 0.001) as assessed by Student’s *t* test

The effect of rapamycin on (hemi)cellulase production of *T. reesei* RUT-C30, an industrial hypercellulolytic strain [30–33], was investigated (Fig. 1). In the presence of 0.01 μM rapamycin, all the tested cellulase activities were not changed at 24 h, but was increased at 72 h and higher than those of RUT-C30 without rapamycin during the rest fermentation time. The pNPGase, pNPCase, CMCase, and FPase activities were improved by 28.7%, 24.0%, 19.2%, and 26.2% separately at 72 h. Nevertheless, when the concentration of rapamycin was increased to 1 μM, the pNPGase, pNPCase, and CMCase activities were reduced at 24 h, but increased at 120 h and beyond. The FPase activity did not reach the same level as that without rapamycin until 168 h. A higher concentration of rapamycin than 1 μM caused a more serious reduction on cellulase activities at the early fermentation process. Especially, the addition of 100 μM rapamycin led to a decline by 67.2%, 60.2%, 56.6%, and 70.3% for pNPGase, pNPCase, CMCase, and FPase activities, respectively, at 24 h. Interestingly, *T. reesei* RUT-C30 had the ability to recover and produce the same or even a larger amount of cellulase at 168 h, regardless of the concentrations of rapamycin. The FPase activity reached ~ 5 IU/mL at 168 h, which was comparable to those (2–8 IU/mL) reported in previous studies under similar culture conditions [34–36]. Obviously, rapamycin improved the cellulase production at low concentration, but at high concentration severely inhibited the cellulase production at the early stage which was restored at the late stage. The secreted protein level followed a similar trend as cellulase activity. The concentration of secreted protein was halved at 100 μM rapamycin at 24 h, as compared to the untreated sample.

The pNPXase activity did not have obvious change at 0.01 μM rapamycin. At 1 μM rapamycin, the pNPXase activity was decreased at 48 h and 72 h, but was restored to the level of that without rapamycin at 120 h. At 10 μM and 100 μM rapamycin, the pNPXase activity was sharply reduced by 68.0% and 71.9% at 24 h, and remained lower than that without rapamycin at 168 h, suggesting that high concentration of rapamycin decreased hemicellulase activity which was unable to be recovered.

A similar effect of 100 μM rapamycin on the (hemi)cellulase production was found in *T. reesei* RUT-C30 grown on glucose or lactose, except that the pNPCase activity was not changed in the presence of rapamycin when using glucose as the carbon source (Additional file 1: Figure S1). Nevertheless, the temporary inhibition effect of rapamycin on the total extracellular protein concentration was not found at the early fermentation stage of *T. reesei* RUT-C30 cultivated on glucose or lactose. In addition, the inhibition effect of rapamycin on the pNPXase activity can be relieved at the late stage, different from that of *T. reesei* RUT-C30 cultured on cellulose. Obviously, the effect of rapamycin on the (hemi)cellulase production in *T. reesei* RUT-C30 was not dependent on the carbon sources, though some subtle differences were observed among different carbon sources.

### Rapamycin induces transit morphology defect of *T. reesei* RUT-C30 on cellulose, but not on lactose or glucose

**Fig. 2:**
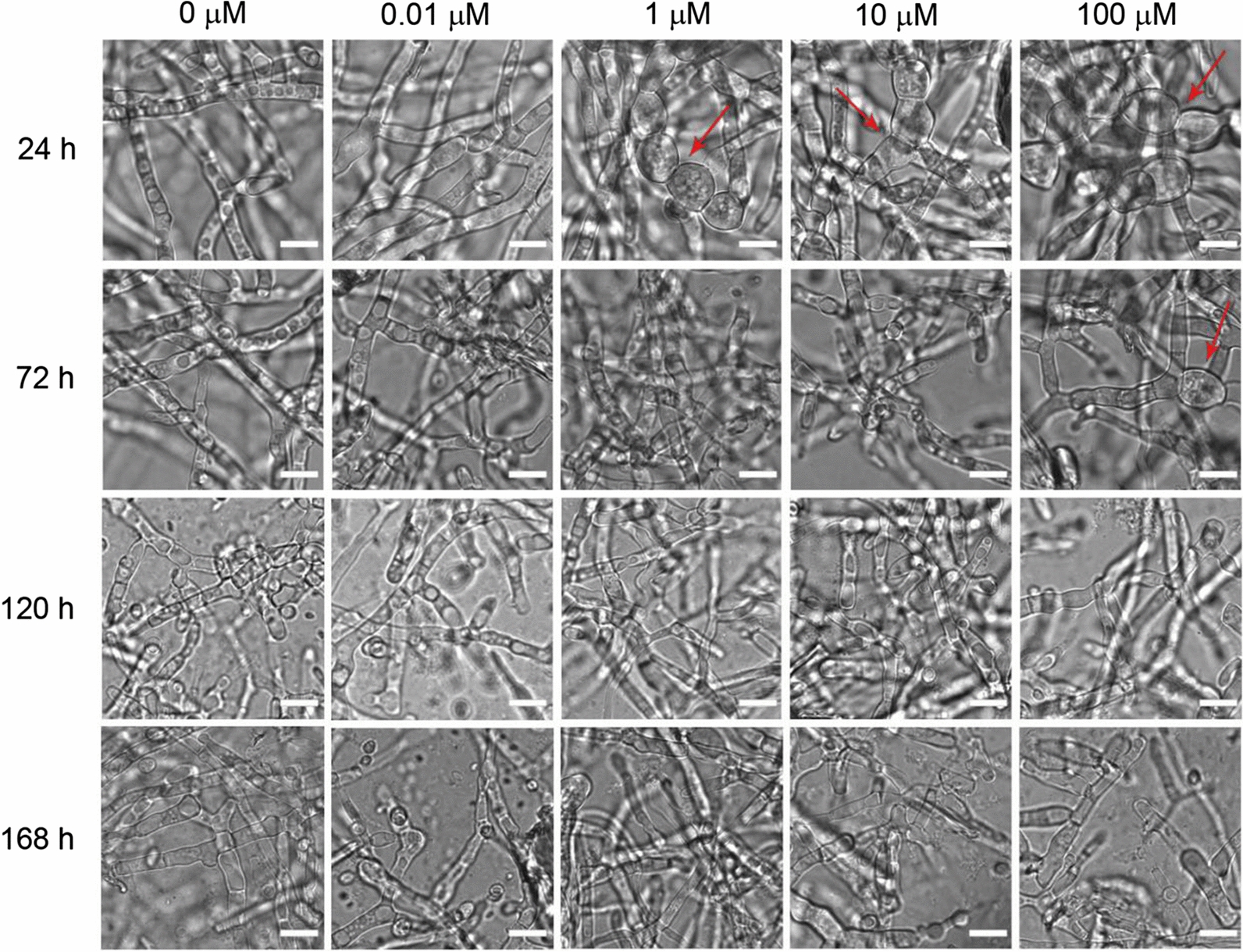
Hyphal morphology of *T. reesei* RUT-C30 cultured in TMM + 2% cellulose with different concentrations of rapamycin at 24 h, 72 h, 120 h, and 168 h, which was observed under CLSM. Scale bar = 10 μm

The effect of rapamycin on the morphology of *T. reesei* RUT-C30 cultivated in TMM + 2% cellulose, 2% lactose or 2% glucose was investigated by confocal laser scanning microscopy (CLSM) (Fig. 2 and Additional file 2: Figure S2). The morphology of *T. reesei* RUT-C30 was not affected by 0.01 μM rapamycin throughout the whole cellulase production on cellulose. When the concentration of rapamycin was increased to 1 μM and beyond, the abnormal hyphal morphology was observed at 24 h. The filamentation of *T. reesei* RUT-C30 was strongly inhibited by rapamycin, showing spherical form which was reminiscent of hypha–yeast transition. However, the morphological defects by 1 μM or 10 μM rapamycin were not found at 72 h and later. Even at 100 μM rapamycin, only a few mycelia exhibited aberrant morphology at 72 h and then disappeared after 72 h. All these findings implicated that rapamycin suppresses the hyphal formation in the early stage of *T. reesei* RUT-C30 grown on cellulose temporarily even at high concentration, which was restored at the late stage. However, when the carbon source was lactose or glucose, there was no notable effect of rapamycin on the mycelium morphology (Additional file 2: Figure S2).

### Rapamycin hinders cell growth and sporulation of *T. reesei* RUT-C30 tentatively, but not the lipid content

**Fig. 3:**
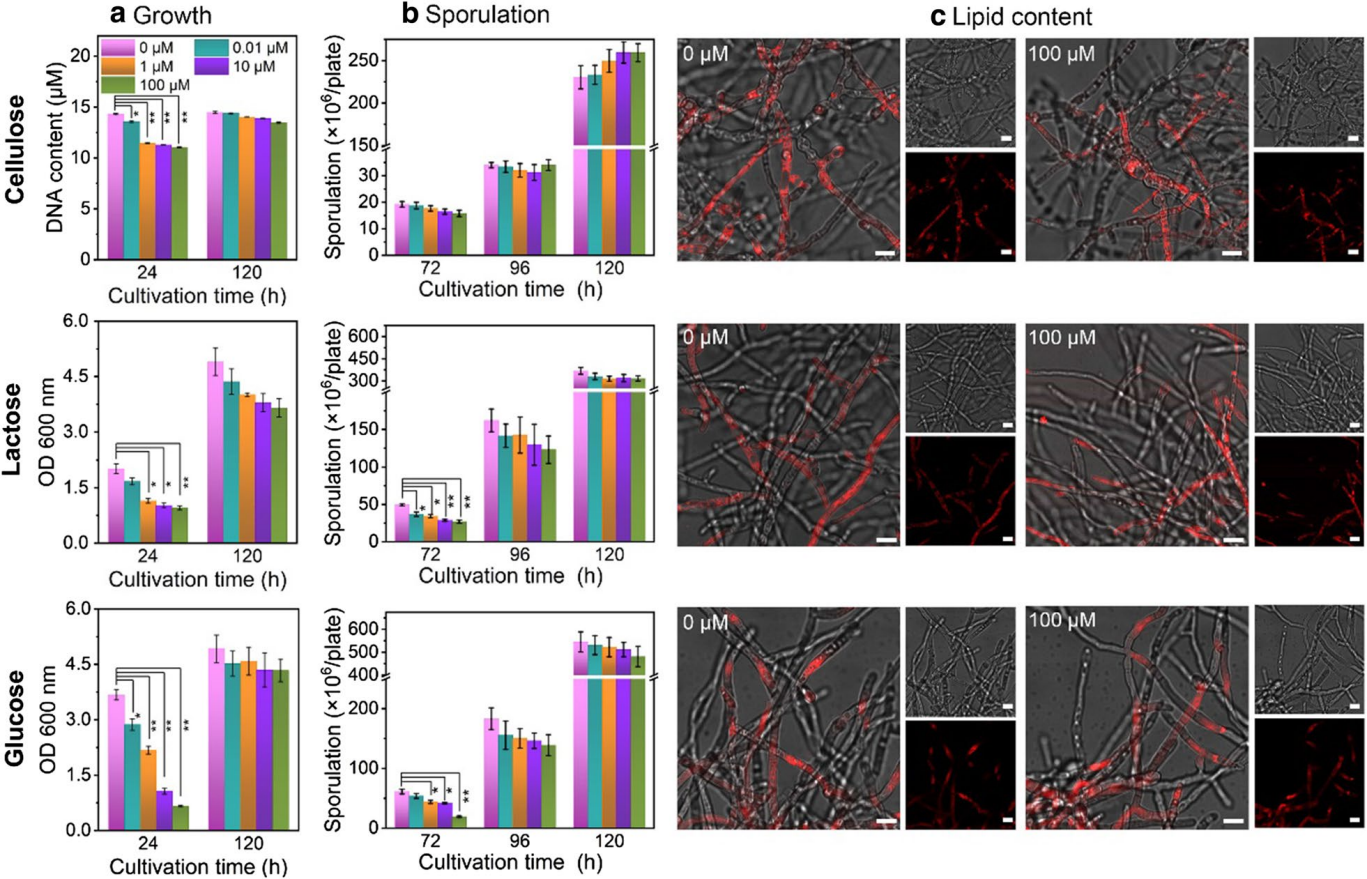
The growth (**a**), sporulation (**b**) and lipid content (**c**) of *T. reesei* RUT-C30 on TMM + 2% cellulose, lactose, or glucose with various concentrations of rapamycin. The lipid content of RUT-C30 at 24 h was stained with Nile Red. Data are represented as the mean of three independent experiments and error bars express the standard. Asterisks indicate significant differences (*p < 0.05, **p < 0.01, ***p < 0.001) as assessed by Student’s *t* test. Scale bar = 10 μm

The effect of rapamycin on the growth of *T. reesei* RUT-C30 on cellulose, lactose or glucose was studied (Fig. 3). Due to the interference of insoluble cellulose to the traditional growth assays like the dry biomass measurement and the optical absorbance assay, the DNA content measurement was utilized to characterize the growth of *T. reesei* on cellulose [7, 34, 37]. As compared to the untreated samples, the cell growth of *T. reesei* RUT-C30 on cellulose, lactose or glucose at 24 h was retarded notably with the treatment of rapamycin at concentrations no less than 1 μM. This cell growth impairment was rescued completely at later stage under cellulose or glucose condition, but not under lactose condition. Meanwhile, the spore amount of *T. reesei* RUT-C30 grown on cellulose, lactose or glucose was also reduced at 24 h, which was recovered at 120 h with no significant change even with 100 μM rapamycin (Fig. 3).

It has been reported that rapamycin treatment led to an increase in the number and size of lipid droplets in the fungus *S. cerevisiae* [38], *Podospora anserina* [39, 40], and *Ustilago maydis* [41]. Therefore, to see whether the lipid content of *T. reesei* RUT-C30 was altered by rapamycin, the lipid content of *T. reesei* RUT-C30 grown on cellulose, lactose or glucose was stained by Nile Red and checked under CLSM (Fig. 3 and Additional file 3: Figure S3). There was no significant difference in the fluorescent intensity and lipid form between rapamycin-treated RUT-C30 and the untreated one, which was independent of the carbon source, implying that the lipid synthesis in *T. reesei* RUT-C30 was not affected by rapamycin. Overall, the cell growth and sporulation of *T. reesei* RUT-C30 were reduced by high-dose rapamycin at the early fermentation stage, but not at the late stage. On the contrary, its lipid content was not influenced by rapamycin. *T. reesei* RUT-C30 is highly resistant to rapamycin regardless of carbon source.

### Transcription pattern of *T. reesei* RUT-C30 treated with high-dose rapamycin

To gain insight into how rapamycin influences *T. reesei* RUT-C30 at the transcriptional level, RNA-seq analysis was performed using RUT-C30 cultured in TMM medium with or without 100 μM rapamycin for 24 h. The sequences of the total reads were mapped to the reference genome of *T. reesei* RUT-C30 (https://www.ncbi.nlm.nih.gov/genome/323%3fgenomeassembly_id%3d49799) with coverage of 97.68–97.76%. A total of 10,048 unique transcripts were detected. Genes were considered to be differentially expressed between the two conditions when the average reads of the corresponding transcripts differed with |log_2_Ratio|≥ 1 and *p* value ≤ 0.05. By comparing rapamycin-treated RUT-C30 to the untreated one, 484 differentially expressed genes (DEGs) were obtained, of which 201 were upregulated and 283 were downregulated (Additional file 4: Table S1).

**Fig. 4:**
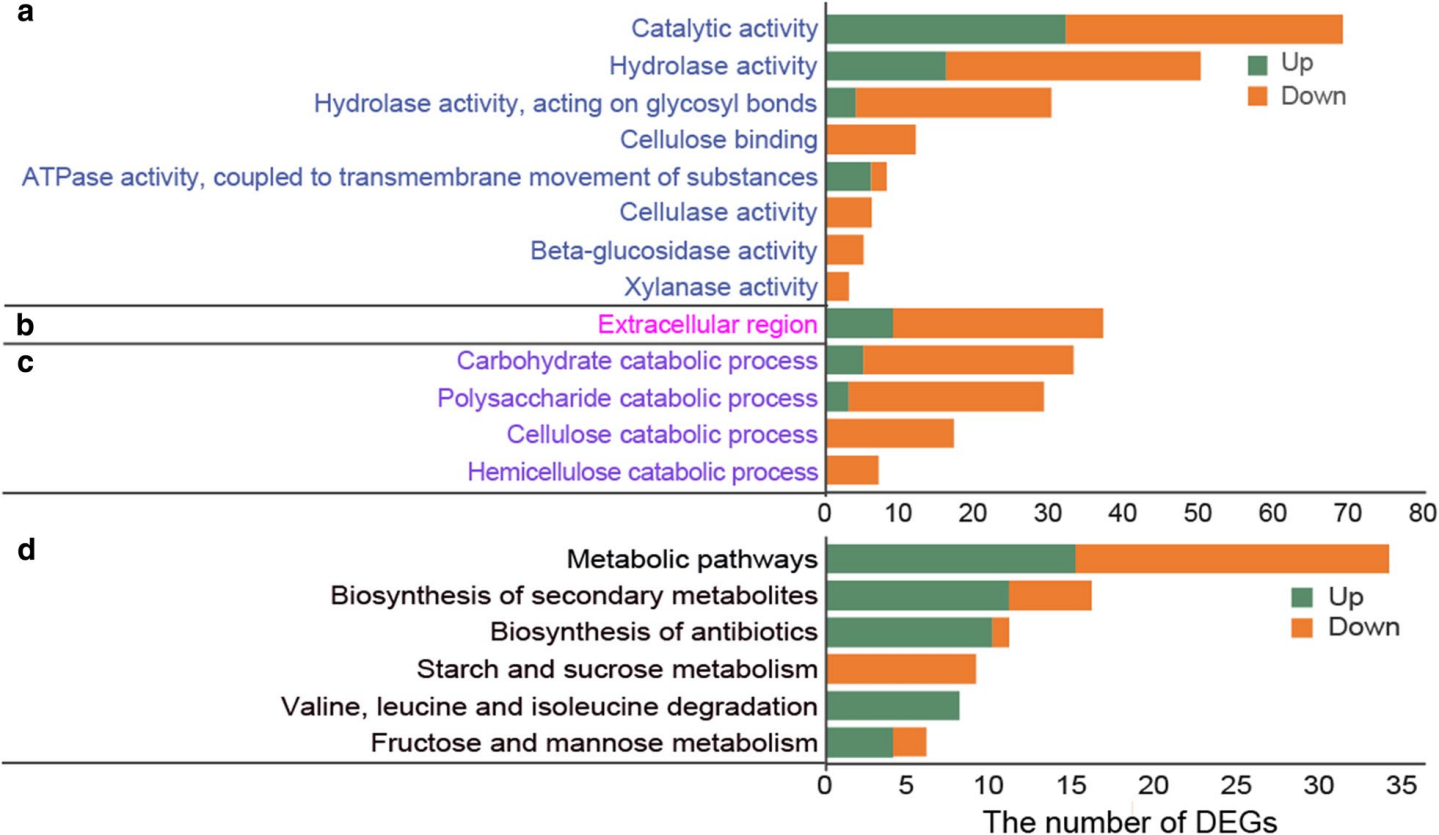
Gene ontology (GO) functional enrichment (**a**–**c**) and Kyoto Encyclopedia of Genes and Genomes (KEGG) enrichment (**d**) analysis of DEGs. **a** The most enriched molecular functions; **b** the most enriched cellular components; **c** the most enriched biological processes; **d** the most enriched pathways. The y-axis represents the name of the most enriched GOs or pathway that belong to different ontologies, while the x-axis represents the number of DEGs in each enriched GO or pathway

The enriched molecular function was mainly related to “catalytic activity” (Fig. 4a), which comprised 69 DEGs. Among them, 50 DEGs show hydrolase activity, of which 30 act on glycosyl bonds. DEGs in “cellulose binding”, “cellulase activity”, “beta-glucosidase activity” and “xylanase activity” categories were all downregulated, which are related to cellulose and hemicellulose degradation. In addition, the category “ATPase activity, coupled to transmembrane movement of substances” included 8 DEGs. Among them, 6 were predicted to be ABC transporters regarding multidrug resistance that were all upregulated to different degrees (Additional file 5: Table S2). The increased expressions of these ABC transporters in *T. reesei* might be a defense mechanism against rapamycin by exporting it out of the cells, which might be worth exploring in future study.

For the enriched cellular components, 37 DEGs were under “extracellular region” category, demonstrating that rapamycin greatly impacts the extracellular enzymes (Fig. 4b). Among them, two cellobiohydrolase (CBH) (CEL7A and CEL6A), seven endoglucanase (EG) (CEL7B, CEL5A, CEL12A, CEL61A, CEL45A, CLE74A, and CEL61B), three β-glucosidase (BGL) (CEL3A, CEL3C and CEL3D), and one β-xylosidase (BXL1) were downregulated. The enriched biological processes were mainly cellulose and hemicellulose catabolic processes, of which all DEGs were downregulated (Fig. 4c). These results were consistent with the remarkably decreased pNPCase, CMCase, pNPGase and pNPXase activities in RUT-C30 treated with 100 μM rapamycin for 24 h, respectively. In addition, the most enriched pathways affected by rapamycin included “Biosynthesis of secondary metabolites”, “Biosynthesis of antibiotic”, “Starch and sucrose metabolism”, “Valine, leucine and isoleucine degradation”, and “Fructose and mannose metabolism” (Fig. 4d).

### DEGs involved in the cellulase production were downregulated by rapamycin

**Fig. 5:**
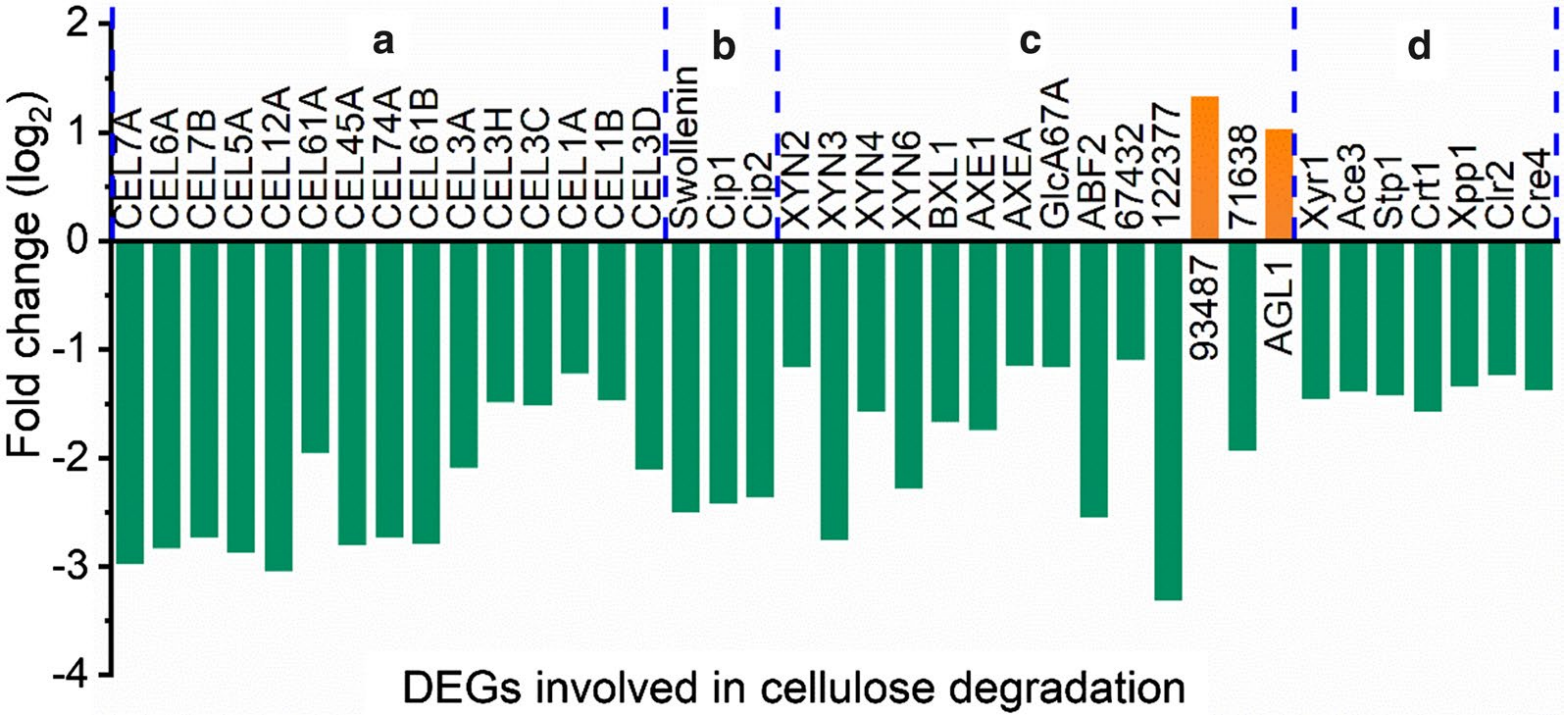
Fold change of DEGs related to cellulose degradation that are categorized into four types: **a** cellulase; **b** nonenzymatic cellulose attacking enzymes; **c** hemicellulase, and **d** transcriptional factors

Thirty-nine DEGs were related to cellulose degradation, of which 37 were downregulated and only two were upregulated (Fig. 5 and Additional file 6: Table S3). Fifteen cellulases including two cellobiohydrolases (CEL7A and CEL6A), seven endoglucanases except CEL5B, and six β-glucosidases (CEL3A, CEL1A, CEL1B, CEL3C, CEL3H and CEL3D) were notably down-expressed, which agreed with the reduced pNPCase, CMCase, pNPGase and total filter paper FPase activities as found above. The downregulated mRNA levels of BXL1 and xylanases (XYN2, XYN3, XYN4, and XYN6) were in line with decreased pNPXase activity. Moreover, nonenzymatic cellulose attacking enzymes swollenin [42], Cip1 and Cip2 [43], which acted in synergy with cellulases and hemicellulases to enhance the hydrolytic efficiency of cellulose were also downregulated significantly. Unexpectedly, the hemicellulase α-galactosidase AGL1 and β-mannosidase (M419DRAFT_93487), which hydrolyses α-D-galactosides and mannans, were upregulated, though another α-galactosidase (M419DRAFT_71638) and two other β-mannosidases (M419DRAFT_67432 and M419DRAFT_122377) were downregulated noticeably. Two lytic polysaccharide monooxygenases (LPMOs), CEL61A (M419DRAFT_139633) and CEL61B (M419DRAFT_122518) were significantly downregulated by 2^1.95^ (3.86)- and 2^2.79^ (6.92)-fold, respectively (Fig. 5). In carbohydrate-active enzymes (CAZymes) database, proteins CEL61A and CEL61B were originally classified as glycoside hydrolase family 61 (GH61), but reclassified as auxiliary activity 9 (AA9) and auxiliary activity 10 (AA10), respectively [44–46]. They have both endoglucanase and LPMO activities [44, 47, 48].

In addition, 7 transcriptional factors involved in cellulase production were identified to be DEGs with marked downregulation (Fig. 5d). They were cellulase transcription activators Xyr1 [49], Ace3 [50] and Clr2 [51], MFS sugar transporters Crt1 and Stp1 [52, 53], xylanase promoter binding protein Xpp1 [54], and the carbon catabolite repressor Cre4 [55]. Xpp1 was firstly described as a repressor of xylanases [56] and later as a repressor of secondary metabolism [54]. The downregulation of Xpp1 did not lead to the increase of xylanase production (Fig. 1), but the expression of 11 DEGs involved in KEGG category “Biosynthesis of secondary metabolites” was found to be increased significantly (Fig. 4d and Additional file 7: Table S4), in agreement with the role of Xpp1 as a repressor of secondary metabolism.

### FKBP12 is required for the temporary inhibition of rapamycin on cellulase production

Inside the cell, rapamycin first forms a gain-of-function complex with FKBP12, the well-known rapamycin cellular receptor [14, 19, 20], which then inhibits the TOR kinases. Currently, FKBP12 has been well studied in both *S. cerevisiae* [22, 57, 58] and human cell [59–61]. It is supposed that *T. reesei* (filamentous fungus) should be more similar to *S. cerevisiae* (a model fungus) than human cells. Therefore, we searched *T. reesei* RUT-C30 genome for the FKBP12 homolog of *S. cerevisiae*, leading to the identification of trFKBP12 (M419DRAFT_72966) hat has already been annotated as FKBP12 in *T. reesei* RUT-C30 genome database published in NCBI or Emsembl Fungi database. Protein trFKBP12 contains 119 amino acids with a predicted molecular mass of 13.3 kDa. Interestingly, the transcription level of trFKBP12 was significantly upregulated by 2^1.71^ (3.27)-fold in *T. reesei* RUT-C30 treated with rapamycin (Table 1), indicating that trFKBP12 is probably the cellular receptor of rapamycin.

**Table 1:** Transcriptional level of FKBP12 and TOR complexes in *T. reesei* RUT-C30

*T. reesei* RUT-C30				*S. cerevisiae*		
Gene ID^a^	Protein ID^b^	log_2_FC	*p* value	Name	Protein ID^b^	Protein identity (%)
M419DRAFT_72966	ETS04725.1	1.71	0.0035	FKBP12	CAA86890	47.32
M419DRAFT_24714	ETS02532.1	− 0.33	5.00E−05	TOR1	NP_012600.1	49.27
				TOR2	NP_012719.2	49.77
M419DRAFT_137337	ETS01798.1	0.11	0.2403	Lst8	NP_014392.3	67.00
M419DRAFT_82078	ETS00934.1	− 0.24	0.0072	Kog1	NP_012056.1	45.92
M419DRAFT_26158	ETR99122.1	0.10	0.2521	Avo1	NP_014563.1	24.23
M419DRAFT_128246	ETS03842.1	− 0.19	0.0194	Avo3	NP_011018.1	26.88

^a^Gene ID was assigned based on the genome database (https://fungi.ensembl.org/Trichoderma_reesei_rut_c_30_gca_000513815/Info/Index)

^b^Protein ID was assigned based on the genome database (https://www.ncbi.nlm.nih.gov/)

**Fig. 6:**
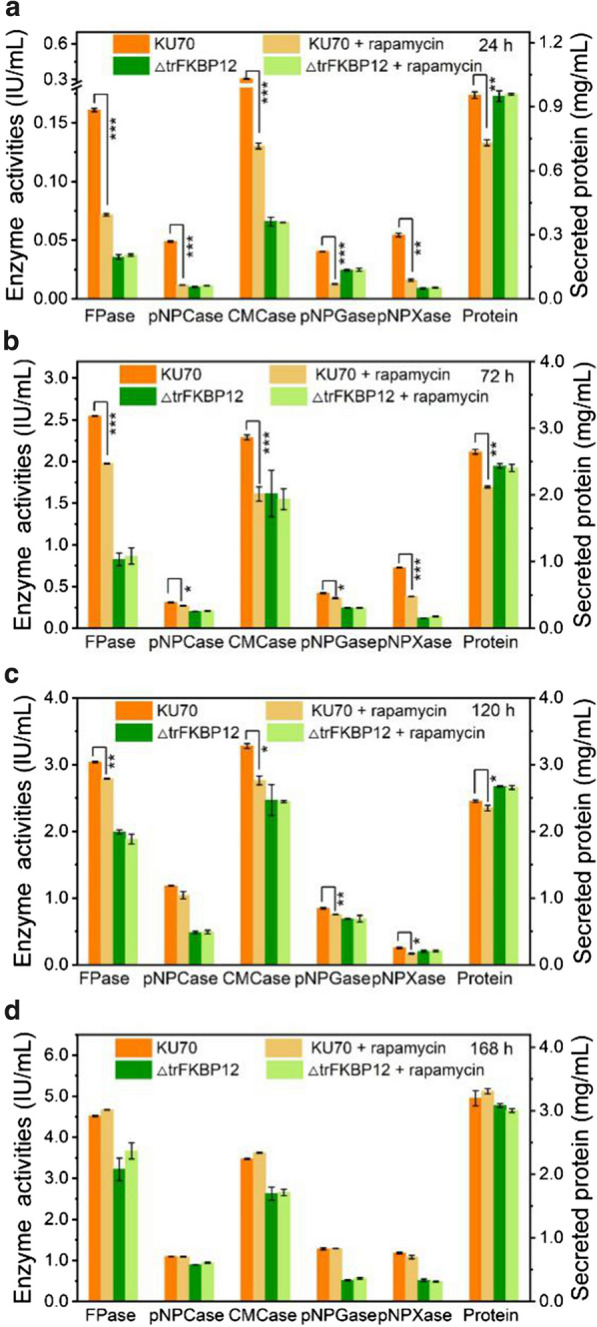
The (hemi)cellulase activities and protein secretion of *T. reesei* KU70 and ΔtrFKBP12 cultured in TMM + 2% cellulose with/ without 100 μM rapamycin at 24 h (**a**), 72 h (**b**), 120 h (**c**) and 168 h (**d**), respectively. FPase: the filter paper activity; pNPCase: the CBH activity; CMCase: the CMC activity; pNPGase: the β-glucosidase activity; pNPXase: the β-xylosidase activity; Protein: secreted protein concentration. Data are represented as the mean of three independent experiments and error bars express the standard. Asterisks indicate significant differences (*p < 0.05, **p < 0.01, ***p < 0.001) as assessed by Student’s *t* test

To determine whether trFKBP12 is the cellular receptor of rapamycin in *T. reesei*, gene *trFKBP12* was knockout using *T. reesei* KU70 as the parent strain [62], obtaining mutant strain ΔtrFKBP12. KU70 was chosen as a host strain for its high efficiency of gene targeting, where *ku70* was deleted in RUT-C30 [63]. Similar to strain RUT-C30, the inhibition effect of 100 μM rapamycin on cellulase production was observed in strain KU70 at the early fermentation stage (Fig. 6a–c), which was completely relieved at the late stage (Fig. 6d). This inhibition effect was not found in strain ΔtrFKBP12 during the whole fermentation process (Fig. 6), demonstrating that trFKBP12 was indispensable for the temporary inhibition effect of rapamycin on cellulase production in *T. reesei* at the early cultivation stage. It is worth noting that knockout of *trFKBP12* alone led to a delay in cellulose production, similar to that caused by high-dose rapamycin. No morphology change was found in strain KU70 or ΔtrFKBP12 with the treatment of 100 μM rapamycin (Additional file 8: Figure S4).

### DEGs involved in the TOR pathway

The TOR kinase is the target of FKBP–rapamycin complex and can interact with multiple proteins to form two complexes TORC1 and TORC2. Both TORC1 and TORC2 play important roles in cell growth and metabolism, but only TORC1 was rapamycin-sensitive [14, 64]. In silico analysis revealed there is only one TOR (M419DRAFT_24714) in *T. reesei*, with 49.27% and 49.77% sequence identity to TOR1 and TOR2 from *S. cerevisiae*, respectively, which was coined trTOR here. The expression of gene *trTOR* was slightly downregulated by rapamycin (Table 1). The essential components of TORC1 (Lst8 and Kog1), and TORC2 (Avo1, Avo3, and Lst8) were identified in *T. reesei*, with little change at transcription levels (Table 1). The other components of the TOR complexes (Tco89, Avo2, and Bit61) were not identified in *T. reesei*. It seems that the effect of rapamycin on the TOR complexes was not very significant in *T. reesei.* The effort to delete gene *trTOR* was failed, indicating that *trTOR* is an essential gene.

Moreover, based on sequence comparisons with the homologues of genes in the TOR signal pathways [14, 65, 66], we identified a series of genes involved in TOR signal pathways in *T. reesei* RUT-C30, including 15 genes in ribosome biogenesis, 8 genes in cell cycle/growth, 25 genes in nutrient uptake, 1 gene in stress, 5 genes in lipid metabolism, 6 genes in cell wall integrity, and 5 genes in autophagy (Additional file 9: Table S5). All these genes were not differentially expressed. These findings matched well with the phenotype profiling results that *T. reesei* RUT-C30 displayed high resistance to rapamycin regardless of carbon sources (Figs. 1, 2, and 3).

### DEGs related to gene expression

Thirty-five out of the total 484 DEGs are related to the gene expression in *T. reesei*, showing that rapamycin has prominent effects on both the transcription and translation level of *T. reesei* RUT-C30 grown on cellulose (Additional file 10: Table S6). DEGs related to DNA metabolism were found, like DNA topoisomerase IV alpha subunit, and DNase I protein. In particular, 14 DEGs are specifically involved in regulation of transcription by RNA polymerase II that is responsible for the transcription of cellulase production [67]. On the other hand, three tRNA were downregulated, including tRNA-Arg, tRNA-Glu and tRNA-Lys, while threonyl/alanyl tRNA synthetase and eukaryotic translation initiation factor 2c were upregulated. In addition, two DEGs function as ribonuclease for RNA degradation. Therefore, 100 μM rapamycin exerted an effect on both the translation and transcription of *T. reesei* RUT-C30 at the early fermentation stage on cellulose.

### DEGs associated with transporters

Forty-seven out of the total 484 DEGs are transporters (Additional file 5: Table S2), including 1 putative efflux pump antibiotic resistance protein, 1 xenobiotic transmembrane transporter, 3 amino acid transporters, 3 aquaporins, 8 ATPase-coupled transmembrane transporters, 10 carboxylate transporters, 11 major facilitator superfamily transporters, 3 transporters for transporting inorganic compounds (1 phosphate permease, 1 acetate transporter, and 1 ammonium permease), 4 transporters for transporting metal ions (1 metal ion transmembrane transporter, 1 siderophore transporter, 1 calcium transmembrane transporter, and 1 zinc ion transmembrane transporter), and 3 other transporters. All the four metal ion transporters were downregulated, while the three amino acid transporters were upregulated.

## Discussion

The cellulase production, cell growth, sporulation ability and lipid content of *T. reesei* RUT-C30 grown on cellulose, lactose or glucose, were not affected by rapamycin even at the high concentration of 100 μM which was much higher than the concentrations inhibiting other organisms. For example, 0.11 μM rapamycin were enough to activate TORC1-controlled transcriptional activators Gln3p, Gat1p, Rtg1p, and Rtg3p and resulted in a fast lipid droplet replenishment in *S. cerevisiae* [38]. Rapamycin at 1.09 μM completely abolished the growth of *P. anserina* [40] and at 0.27 μM induced autophagy in hyphae of *F. graminearum* [21]. Obviously, *T. reesei* RUT-C30 is highly resistant to rapamycin, which is independent of carbon sources. The resistance to rapamycin were also observed in *S. pombe* [68], and land plants like *Arabidopsis thaliana*, the bryophyte *Physcomitrella patens*, the monocot *Oryza sativa*, and the dicots *Nicotiana tabacum* and *Brassica napus* [16], though rapamycin susceptibility is widespread among eukaryotes, inhibiting the growth of most fungi and animal cells [38, 69]. The reason why *T. reesei* RUT-C30 is insensitive to rapamycin is still unknown.

It has been reported that mutations of *FKBP12* prevent the formation of FKBP–rapamycin complex, and mutations in the FRB (FKPB12–rapamycin-binding) domain of TOR1 block the binding of FKBP–rapamycin to TOR1, both conferring rapamycin resistance. Therefore, we performed sequence alignments of rapamycin-binding domains of trTOR and trFKBP12 with those of the corresponding homologs from organisms that are sensitive (*S. cerevisiae* and *Homo sapiens*) or insensitive (*A. thaliana*, *S. pombe*, and *O. sativa*) to rapamycin (Additional file 11: Figure S5). Most of the amino acids required for the formation of hydrophobic rapamycin-binding pocket [70] are well conserved in trFKBP12, including Tyr^31^, Phe^48^, Ile^68^, Trp^71^, Tyr^94^, Ile^103^ and Phe^111^ (numbered according to trFKBP12) (Additional file 11: Figure S5A). Similarly, the amino acid residue Trp^2101^, Phe^2108^, or Ser^2035^ of mTOR (mammalian target of rapamycin), mutation of which confers rapamycin resistance [71], is also present in trTOR (Additional file 11: Figure S5B). These findings demonstrate that the high resistance of *T. reesei* to rapamycin was not due to mutations of trFKBP12 and trTOR on comparison to counterparts from organisms that are susceptible to rapamycin.

The temporary inhibition effect of rapamycin on *T. reesei* RUT-C30 might due to the degradation of rapamycin either chemically or by RUT-C30 during the culture process. Currently, it is unknown how stable rapamycin is under our cultivation conditions, which is worthy of further study. As far as we know, no rapamycin degradation pathway in *T. reesei* has been reported. The hyphal growth of *Verticillium dahliae* hypersensitive to rapamycin was severely inhibited in the presence of 0.2 μM rapamycin even after 11 days’ cultivation [72]. Moreover, when firstly cultured in TMM medium for 96 h and treated with different concentrations of rapamycin for 24 h, the (hemi)cellulase activities and protein secretion of *T. reesei* RUT-C30 were not affected by rapamycin (Additional file 12: Figure S6). It seems that the temporary inhibition effect on RUT-C30 by rapamycin only occurs at the early fermentation stage. In addition, rapamycin can bind to ABC transporters with high affinity, acting as a substrate for transport by ABC transporters out of cells and causing rapamycin resistance [73]. Rapamycin biosynthetic gene cluster in rapamycin-producing strains *S. hygroscopicus* and *Streptomyces rapamycinicus* contains putative ABC transporter genes (*OrfX* and *rapX*) to prevent self-poisoning by exporting rapamycin from inside cells [74, 75]. In *T. reesei* RUT-C30, 8 ABC transporters were significantly changed at mRNA level (Additional file 5: Table S2), of which 6 transporters related to multidrug resistance were upregulated markedly, including M419DRAFT_75459, M419DRAFT_90404, M419DRAFT_109180, M419DRAFT_116127, M419DRAFT_97054 and M419DRAFT_114328 (Additional file 5: Table S2). The noticeable increase of these ABC transporters might contribute to the recovery of enzymatic activities at later fermentation process in *T. reesei* RUT-C30 and its rapamycin resistance. Taken together, we believe that the temporary inhibition effect of rapamycin on *T. reesei* RUT-C30 at the early fermentation stage should not be due to rapamycin degradation during cultivation.

*T. reesei* RUT-C30 might represent an excellent platform to study the resistance of cells to rapamycin which is commonly countered when rapamycin is explored as antifungal or anticancer agents. mTOR has become a focus for cancer drug development [76]. Rapamycin is a highly specific inhibitor of mTOR and potently suppresses tumor cell growth by retarding cells in G1 phase or potentially inducing apoptosis. Currently, rapamycin and its analogues are being evaluated as anticancer agents in clinical trials. However, many human cancers have displayed intrinsic resistance or acquired resistance to rapamycin [77]. Though several predicted mechanisms behind the resistance of cancer cells to rapamycin have been proposed, the detailed mechanisms remain to be explored. The high rapamycin resistance of *T. reesei* RUT-C30 might endow it as a model for the eukaryotic cell to unravel mechanism of rapamycin resistance.

The expression level of gene *trFKBP12* was remarkably increased in the presence of high-concentration rapamycin. In *Fusarium fujikuroi* and *Botrytis cinerea*, rapamycin treatment also led to an increased transcription level of FKBP12-encoding gene *fpr1* [78, 79]. Knockout of gene *trFKBP12* caused a transient inhibition effect on cellulase production at the early fermentation stage (Fig. 6), as compared to the parent strain KU70. Furthermore, *trFKBP12* knockout completely abolished the inhibition effect of high-dose rapamycin on cellulase production in *T. reesei,* as indicated by the unchanged cellulase production in strain ΔtrFKBP12 treated with high-dose rapamycin. These findings imply that the temporary inhibition of rapamycin on cellulase requires trFKBP12, and free trFKBP12 might play a role in the initial production of cellulase. Both the deletion of trFKBP12 and the addition of rapamycin can result in a reduced level of free trFKBP12. Rapamycin can bind trFKBP12 to form the rapamycin–trFKBP12 complex, leading to the reduction of free trFKBP12. In contrast to the profound upregulation of trFKBP12, the expression of trTOR was only slightly downregulated and genes involved in the TOR signaling pathways were not changed markedly, which might be associated with the high rapamycin resistance in *T. reesei*. FKBP12 mediated rapamycin sensitivity via TOR in most eukaryote cells [80–82].

Despite that *T. reesei* exhibited high resistance to rapamycin, its cellulase production, cell growth and sporulation were inhibited by high concentration of rapamycin at the early fermentation stage, which was independent of carbon sources and was relieved at the late stage. In addition, the presence of high-concentration rapamycin induced morphology defect in strain RUT-C30 at the early cultivation on cellulose. However, this morphology defect was not found in RUT-C30 grown on glucose or lactose, and strain KU70 or ΔtrFKBP12 propagated on cellulose, indicating that it is a very specific phenotype for RUT-C30 cultured on cellulose. Similar hyphal morphology alterations with the treatment of rapamycin were previously reported in other filamentous fungi like *P. anserina* [40] and *U. maydis* [83].

## Conclusion

In this study, we have investigated the effect of rapamycin on cellulase production, filamentous morphology, cell growth, sporulation, and lipid content of *T. reesei* RUT-C30 grown on different carbon sources (cellulose, lactose and glucose). The high-dose rapamycin (100 μM) induced a delay in cellulase production, cell growth and sporulation regardless of the carbon sources and specifically caused morphology defect in RUT-C30 grown on cellulose, both of which were transient and restored at the late fermentation stage. In contrast, the lipid content of *T. reesei* RUT-C30 cultivated on cellulose, lactose or glucose was not changed by rapamycin. These findings implicate that *T. reesei* RUT-C30 is highly resistant to rapamycin. In line with the phenotype profiling results, transcriptomic analysis found that the mRNA levels of genes associated with the cellulase production were decreased severely by rapamycin. Furthermore, the transcriptional level of the putative rapamycin receptor trFKBP12 was increased significantly, while those of gene *trTOR* (the downstream effector of the rapamycin–FKBP12 complex) and genes associated with the TOR signaling pathways were not changed markedly, matching well with the super rapamycin resistance of *T. reesei* we observed. Upon the deletion of gene *trFKBP12*, there is no influence of rapamycin on cellulase production, indicating the transient inhibition of high-dose rapamycin on cellulase production is assisted by trFKBP12. Overall, we first discovered that *T. reesei* is highly resistant to rapamycin, probably owing to that trTOR and its relevant signaling pathways were not very sensitive to rapamycin. These results deepen our understanding of the impact of rapamycin and the role of the TOR signaling pathways in *T. reesei,* a cellulose-producing workhouse in industry.

## Methods

### Microbial strains, plasmids and cultivation conditions

*Escherichia coli* DH5α was explored for propagation and construction of plasmids. *Agrobacterium tumefaciens* AGL-1 was used as a T-DNA donor for fungal transformation. *T. reesei* RUT-C30 (CICC 13052, ATCC 56765) was purchased from China Center of Industrial Culture Collection. *T. reesei* KU70, derived from *T. reesei* RUT-C30 by deleting gene *ku70*, was provided friendly by Professor Wei Wang from East China University of Science and Technology [84]. Plasmid pXBthg was a gift from Professor Zhihua Zhou from Key Laboratory of Synthetic Biology, Shanghai [85]. *E. coli* DH5α and *A. tumefaciens* AGL-1 were cultivated in Luria–Bertani (LB) with 220 rpm at 37 °C and 28 °C, respectively. *T. reesei* were grown on potato dextrose agar (PDA) plates at 28 °C for conidia production and in *Trichoderma* minimal media (TMM) [86] with 2% (w/t) cellulose, lactose or glucose at 28 °C with 220 rpm. TMM was a well-used medium for *T. reesei* cultivation for cellulase production [8, 34, 35, 37, 86, 87]. Cellulose and lactose are insoluble and soluble inducers for cellulase production, respectively, while glucose represses cellulase production. All chemicals used in this research were ordered from Sigma-Aldrich, USA.

### Shake-flask cultivation

Five percent (v/v) 10^7^/mL conidia of *T. reesei* RUT-C30 were inoculated into 10 mL (Sabouraud dextrose broth) SDB and incubated at 28 °C with 220 rpm for 2 days. Ten percent (v/v) pre-grown mycelia were inoculated into 50 mL TMM media (pH 6) with different concentrations of rapamycin as indicated in the text using 2% cellulose, lactose, or glucose as the carbon source, and then incubated at 28 °C with 200 rpm for 7 days. The stock solution of rapamycin (50 mM) was prepared in DMSO with sonication. Samples were taken at varied time points as indicated in the context for (hemi)cellulase activity assay, confocal observation of fungal mycelia, biomass measurement, Nile Red staining and RNA-seq analysis. If necessary, samples were centrifuged at 8000 rpm for 30 min to separate the mycelia and the supernatant.

### Analysis method

(Hemi)cellulase activities, confocal observation of fungal mycelia, DNA content measurement, sporulation assay and RNA-seq analysis were performed as described in our previous research [34, 37, 87]. The growth of strain *T. reesei* grown in TMM + 2% cellulose with and without rapamycin was assayed by DNA content measurement, while by measuring the optical density of the culture suspension at 600 nm (OD 600 nm) with a UV–vis spectrophotometer (UV-2600, Shimadzu, Japan) when cultured in TMM + 2% lactose or TMM + 2% glucose.

### Lipid content analysis by Nile Red staining

For Nile Red staining, fresh mycelia collected as above were washed with PBS for two times, and incubated with Nile Red solution (25 ng/mL) at 28 °C for 10 min. Then, the samples were washed with PBS for two times, and checked under a confocal microscope (TCS SP8, Leica, Germany) with the excitation wavelength of 510–560 nm and the emission wavelength of 600–680 nm.

### Deletion of gene trFKBP12 in *T. reesei* KU70

The upstream and downstream sequences (~ 1500 bp) of gene *trFKBP-12* were amplified separately by PCR using genomic DNA of *T. reesei* KU70 as a template, and cloned into plasmid pXBthg at XhoI and at BamHI, respectively, using ClonExpress™ II One Step Cloning Kit (Vazyme, China), leading to plasmid pXBthg-FKBP. The resulting plasmid pXBthg-FKBP was transformed into *T. reesei* KU70 by the *Agrobacterium tumefaciens*-mediated transformation (AMT) method [88]. Sixty hygromycin-resistant transformants per 10^7^ conidia were obtained on PDA medium with 50 μg/mL hygromycin, of which 3 transformants were selected randomly for three consecutive subcultures to achieve genetic stability. Then genome DNA of these three transformants was extracted and used as template for PCR verification. The PCR verification was performed using 2*Phanta Max Master Mix (Vazyme, China) with the following PCR reaction conditions: 95 °C for 3 min; 35 cycle of 95 °C for 15 s, 67 °C for 15 s and 72 °C for 210 s; 72 °C for 5 min. All three transformants were proved to be trFKBP12-deleted (Additional file 13: Figure S7), of which one was further confirmed by sequencing at Sangon Biotech and named as strain ΔtrFKBP12. The primers used are listed in Additional file 14: Table S7.

## Supplementary Information

**Additional file 1: Figure S1.** Effects of 100 μM rapamycin on (hemi)cellulase activities and protein secretion of *T. reesei* RUT-C30 cultured in TMM + 2% glucose (A) and TMM + 2% lactose (B) at 24 h, 72 h, 120 h, and 168 h, respectively. FPase: the filter paper activity; pNPCase: the CBH activity; CMCase: the CMC activity; pNPGase: the β-glucosidase activity; pNPXase: the β-xylosidase activity; Secreted protein: secreted protein concentration. Data are represented as the mean of three independent experiments and error bars express the standard. Asterisks indicate significant differences (*p < 0.05, **p < 0.01, ***p < 0.001) as assessed by Student’s *t* test.

**Additional file 2: Figure S2.** Hyphal morphology of *T. reesei* RUT-C30 cultured in TMM + 2% glucose (A) or lactose (B) with different concentrations of rapamycin at 24, 72, 120, and 168 h, which was observed under CLSM. Scale bar = 10 μm.

**Additional file 3: Figure S3.** The lipid content of *T. reesei* RUT-C30 on TMM + 2% cellulose, lactose, or glucose with various concentrations of rapamycin. The lipid content of RUT-C30 at 24 h stained with Nile Red was observed under CLSM. Scale bar = 10 μm.

**Additional file 4: Table S1.** Transcriptional level of total DEGs in *T. reesei* RUT-C30 with or without 100 μM rapamycin.

**Additional file 5: Table S2.** Transcriptional level of DEGs related to transporters in *T. reesei* RUT-C30.

**Additional file 6: Table S3.** Transcriptional level of genes involved in (hemi)cellulase production in *T. reesei* RUT-C30.

**Additional file 7: Table S4.** Transcriptional level of DEGs related to “Biosynthesis of secondary metabolites” in *T. reesei* RUT-C30.

**Additional file 8: Figure S4.** Hyphal morphology of *T. reesei* △FKBP12 and KU70 cultured in TMM + 2% cellulose with/without 100 μM rapamycin at 24, 72, 120, and 168 h, which was observed under CLSM. Scale bar = 20 μm.

**Additional file 9: Table S5.** Transcriptional level of genes involved in TOR signal pathways in *T. reesei* RUT-C30.

**Additional file 10: Table S6.** Transcription level of genes associated with gene expression in *T. reesei* RUT-C30.

**Additional file 11: Figure S5.** FKBP12 (A) and TOR (B) sequence alignments with those of *Saccharomyces cerevisiae*, *Homo sapiens*, *Arabidopsis thaliana*, *Schizosaccharomyces pombe*, and *Oryza sativa.* Amino acid residues that form the hydrophobic rapamycin-binding pocket of FKBP12 (A) are in red. Mutation of amino acid residues of TOR (B) in red confers rapamycin resistance.

**Additional file 12: Figure S6.** The (hemi)cellulase activities and protein secretion of *T. reesei* RUT-C30 cultured in TMM + 2% cellulose for 96 h and treated with different concentrations of rapamycin for 24 h. FPase: the filter paper activity; pNPCase: the CBH activity; CMCase: the CMC activity; pNPGase: the β-glucosidase activity; pNPXase: the β-xylosidase activity; Secreted protein: secreted protein concentration. Data are represented as the mean of three independent experiments and error bars express the standard. Asterisks indicate significant differences (*p < 0.05, **p < 0.01, ***p < 0.001) as assessed by Student’s *t* test.

**Additional file 13: Figure S7.** Gene *trFKBP12* was deleted in *T. reesei* KU70 by homologous integration (A) to obtain putative ΔtrFKBP12 strains (1, 2 and 3) in which the successful deletion of *trFKBP* were verified by PCR (B). FKBP, *trFPBP12*; hyg, hygromycin B phosphotransferase; M: DL5000 marker; Primer 1: Primer 1-F and Primer 1-R; Primer 2: Primer 2-F and Primer 2-R.

**Additional file 14: Table S7.** Primers for *trFKBP* deletion and confirmation.

## Data Availability

The datasets supporting the conclusions of this article are included in the article and its Additional files.

## Abbreviations

TOR: Target of rapamycin
BGL: β-Glucosidase
EG: Endoglucanase
CBH: Cellobiohydrolase
GH: Glycoside hydrolase
pNPGase: The β-glucosidase activity
pNPCase: The CBH activity
CMCase: The CMC activity
FPase: The filter paper activity
pNPXase: The β-xylosidase activity
AMT: *Agrobacterium*-Mediated fungal transformation
SDB: Sabouraud dextrose broth
TMM: *Trichoderma* minimal medium
PDA: Potato dextrose agar
