# Supplementary figures and images for: High-dose rapamycin exerts a temporary impact on T. reesei RUT-C30 through gene trFKBP12
Source: Biotechnol Biofuels. 2021 Mar 26;14:77. doi: 10.1186/s13068-021-01926-w (PMC8004424; doi:10.1186/s13068-021-01926-w)

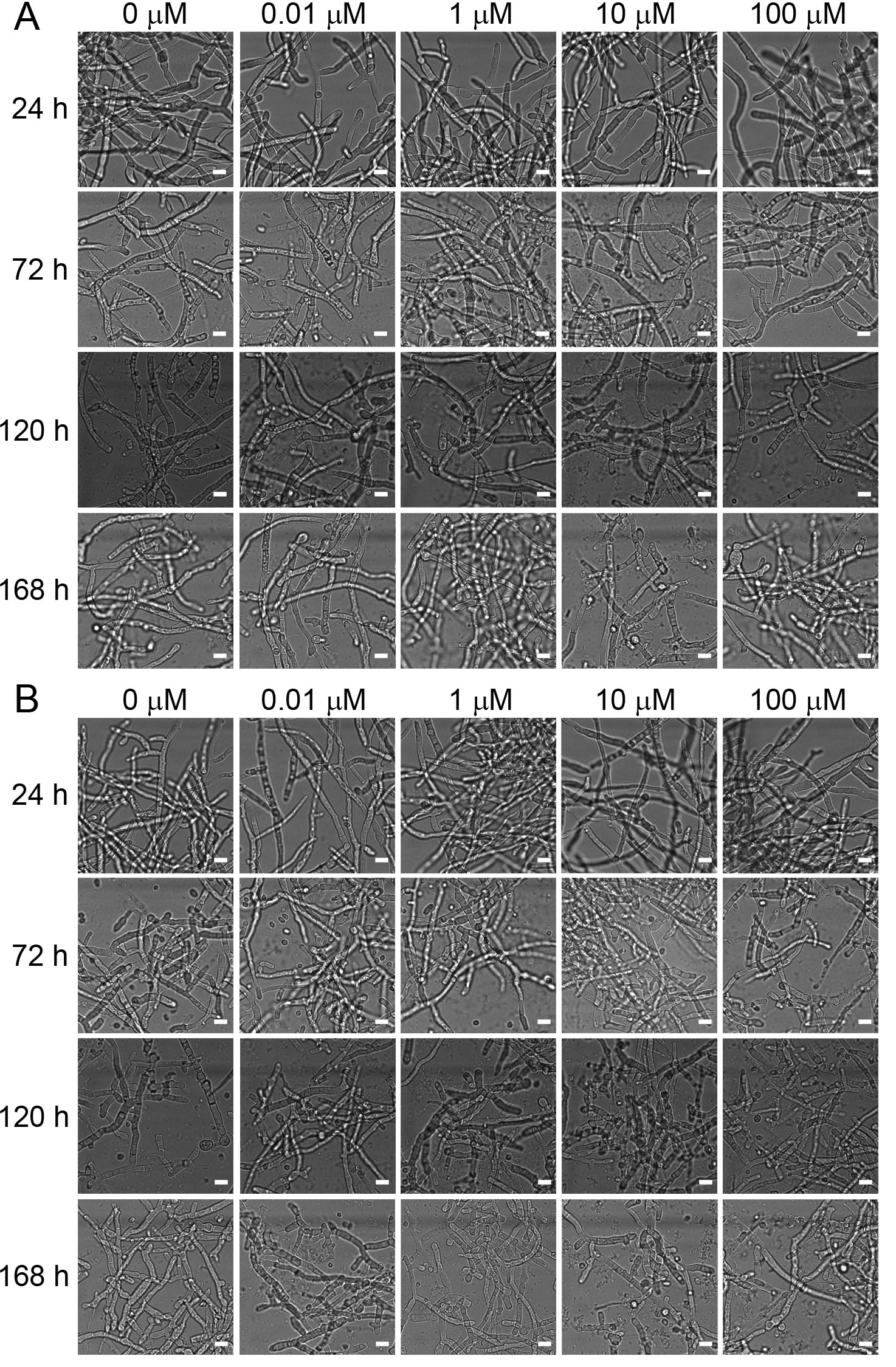

Supplement: Supplementary file 2 — Additional file 2: Figure S2. Hyphal morphology of T. reesei RUT-C30 cultured in TMM + 2% glucose (A) or lactose (B) with different concentrations of rapamycin at 24, 72, 120, and 168 h, which was observed under CLSM. Scale bar = 10 μm. [file 13068_2021_1926_MOESM2_ESM.jpg]

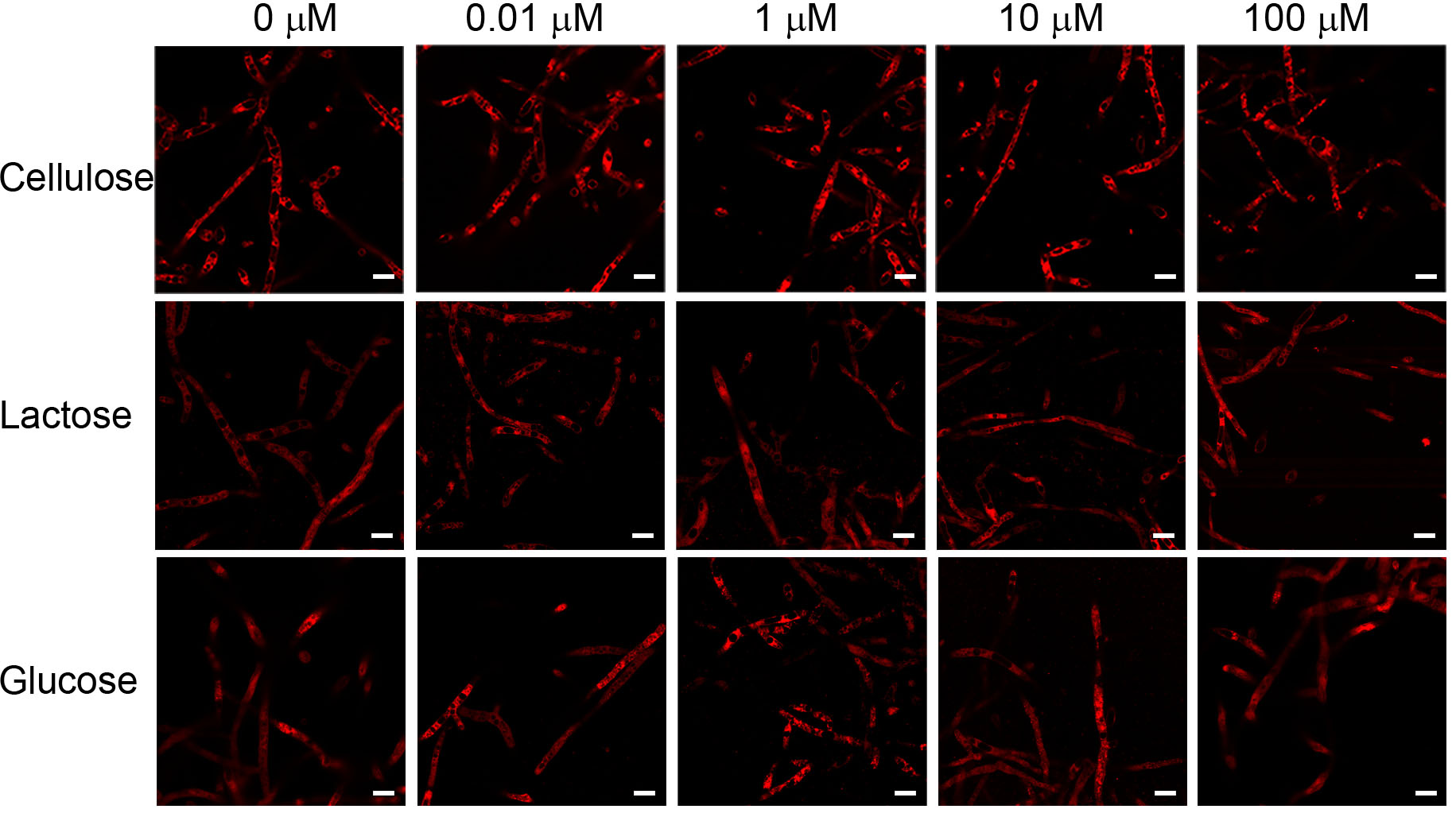

Supplement: Supplementary file 3 — Additional file 3: Figure S3. The lipid content of T. reesei RUT-C30 on TMM + 2% cellulose, lactose, or glucose with various concentrations of rapamycin. The lipid content of RUT-C30 at 24 h stained with Nile Red was observed under CLSM. Scale bar = 10 μm. [file 13068_2021_1926_MOESM3_ESM.jpg]

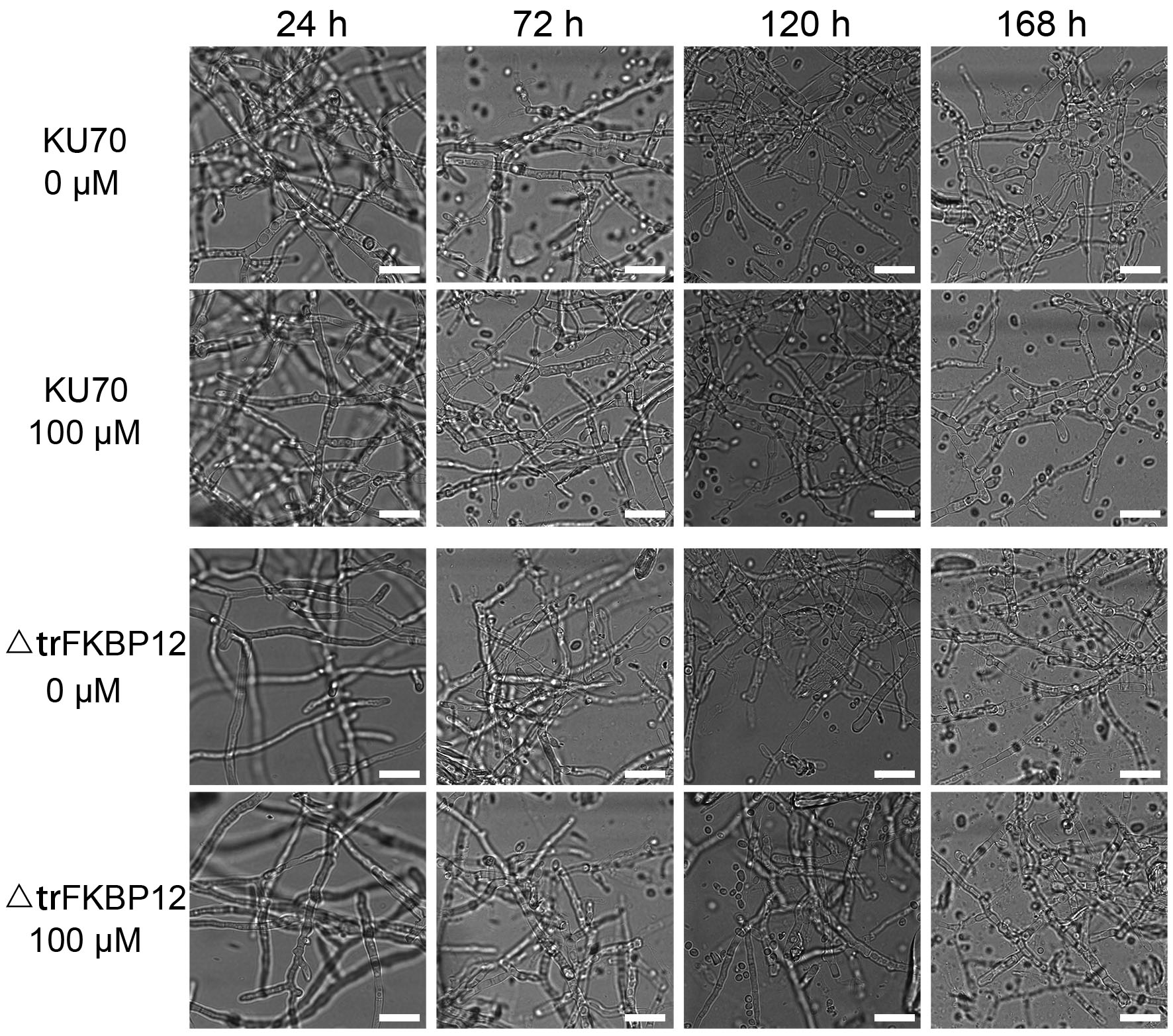

Supplement: Supplementary file 8 — Additional file 8: Figure S4. Hyphal morphology of T. reesei △FKBP12 and KU70 cultured in TMM + 2% cellulose with/without 100 μM rapamycin at 24, 72, 120, and 168 h, which was observed under CLSM. Scale bar = 20 μm. [file 13068_2021_1926_MOESM8_ESM.jpg]

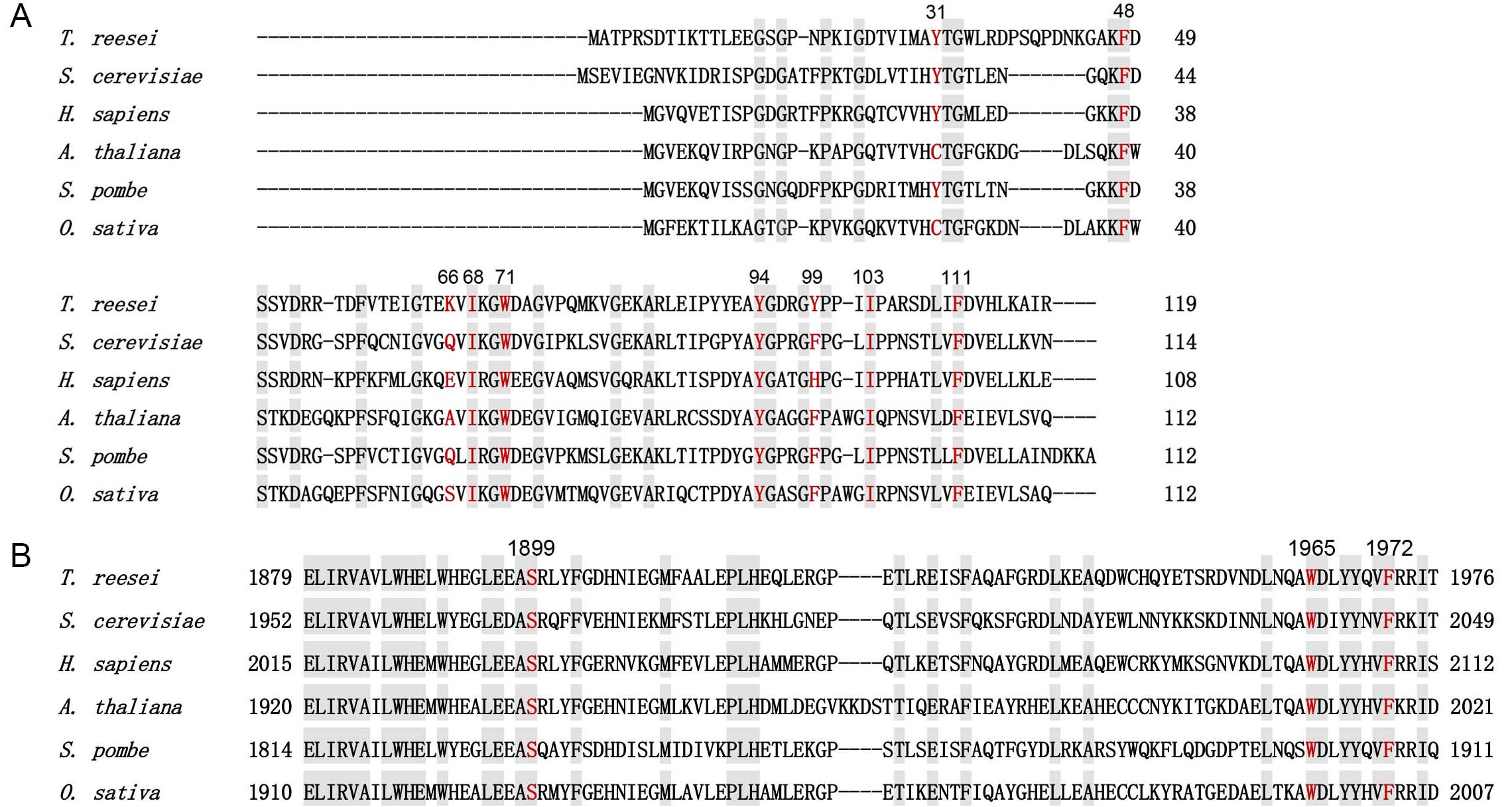

Supplement: Supplementary file 11 — Additional file 11: Figure S5. FKBP12 (A) and TOR (B) sequence alignments with those of Saccharomyces cerevisiae, Homo sapiens, Arabidopsis thaliana, Schizosaccharomyces pombe, and Oryza sativa. Amino acid residues that form the hydrophobic rapamycin-binding pocket of FKBP12 (A) are in red. Mutation of amino acid residues of TOR (B) in red confers rapamycin resistance. [file 13068_2021_1926_MOESM11_ESM.jpg]

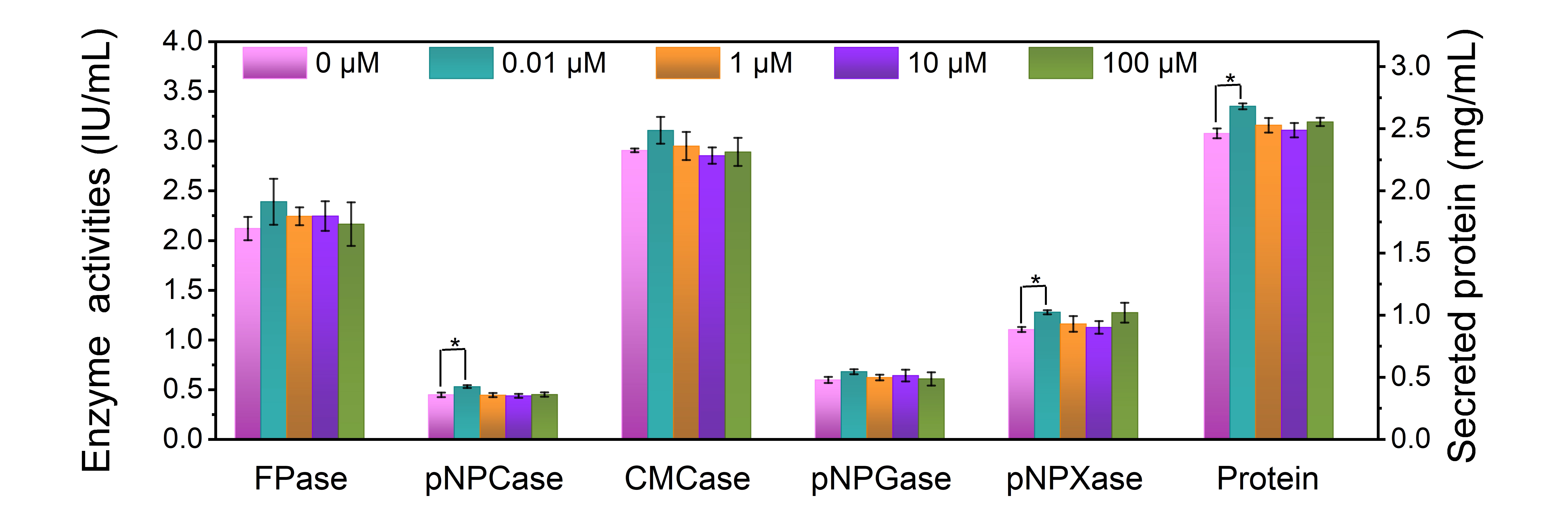

Supplement: Supplementary file 12 — Additional file 12: Figure S6. The (hemi)cellulase activities and protein secretion of T. reesei RUT-C30 cultured in TMM + 2% cellulose for 96 h and treated with different concentrations of rapamycin for 24 h. FPase: the filter paper activity; pNPCase: the CBH activity; CMCase: the CMC activity; pNPGase: the β-glucosidase activity; pNPXase: the β-xylosidase activity; Secreted protein: secreted protein concentration. Data are represented as the mean of three independent experiments and error bars express the standard. Asterisks indicate significant differences (*p < 0.05, **p < 0.01, ***p < 0.001) as assessed by Student’s t test. [file 13068_2021_1926_MOESM12_ESM.jpg]

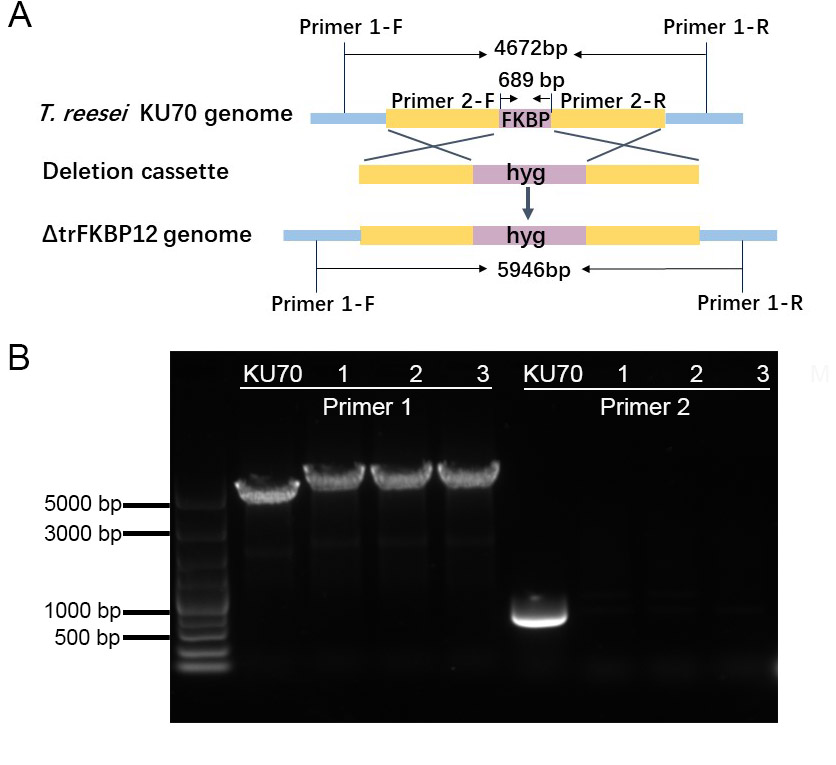

Supplement: Supplementary file 13 — Additional file 13: Figure S7. Gene trFKBP12 was deleted in T. reesei KU70 by homologous integration (A) to obtain putative ΔtrFKBP12 strains (1, 2 and 3) in which the successful deletion of trFKBP were verified by PCR (B). FKBP, trFPBP12; hyg, hygromycin B phosphotransferase; M: DL5000 marker; Primer 1: Primer 1-F and Primer 1-R; Primer 2: Primer 2-F and Primer 2-R. [file 13068_2021_1926_MOESM13_ESM.jpg]
