# Supplementary material for: High-dose rapamycin exerts a temporary impact on T. reesei RUT-C30 through gene trFKBP12
Source: Biotechnol Biofuels. 2021 Mar 26;14:77. doi: 10.1186/s13068-021-01926-w (PMC8004424; doi:10.1186/s13068-021-01926-w)
Supplement: Supplementary file 14 — Additional file 14: Table S7. Primers for trFKBP deletion and confirmation. [file 13068_2021_1926_MOESM14_ESM.docx]

Additional file 1：

Table S7 Primers for *trFKBP-12* deletion and confirmation

| Primer | Sequence (5’– 3’) |
| --- | --- |
| **For *trFKBP-12* deletion** | |
| FKBP-UP-F | ATTATTATGGAGAAACTCGAGAGCAACCGGCACGGATCACCAA |
| FKBP-UP-R | CCGTCACCAGCCCTGCTCGAGTTTTGACGAACGTTGAAGTATCCCG |
| FKBP-DO-F | GTGAGGGTTAATTGCGCGGATCCGACGTGCGATCCGGATACGA |
| FKBP-DO-R | CAGGTCGACTCTAGAGAGGATCCCGAAAGATCCGCAATCTGCACC |
|  | |
| **For PCR confirmation of *T. reesei* ΔtrFKBP12 mutants** | |
| Primer 2-F | ATGGCCACCCCTCGGAGCGA |
| Primer 2-R | CTATCGGATCGCCTTCAGGTGGAC |
| Primer 1-F | GCTGTCCGAACTTCTTCTCTGACG |
| Primer 1-R | TCCATGCCCTCGATTTCGGAGA |
